# Refining Chemotherapy Decisions in Early-Stage Breast Cancer: A Comparison of MammaPrint and National Comprehensive Cancer Network (NCCN) Criteria in a Bosnian and Herzegovinian Cohort

**DOI:** 10.7759/cureus.82825

**Published:** 2025-04-23

**Authors:** Vedad Dedic, Sadat Pusina, Mirhan Salibasic, Emina Bicakcic-Filipovic, Emir Bicakcic, Naida Herenda, Nidzara Karup

**Affiliations:** 1 Department of General, Abdominal and Glandular Surgery, University Clinical Center of Sarajevo, Sarajevo, BIH; 2 Department of Oncology, University Clinical Center of Sarajevo, Sarajevo, BIH; 3 Department of Anaesthesiology, University Clinical Center of Sarajevo, Sarajevo, BIH; 4 Department of Otolaryngology, General Hospital "Prim.dr Abdulah Nakas", Sarajevo, BIH

**Keywords:** adjuvant chemotherapy, breast neoplasm, genetic profiling, mammaprint, risk assessment

## Abstract

Background

Adjuvant chemotherapy decisions in early-stage, hormone receptor-positive, HER2-negative breast cancer traditionally rely on clinicopathological features such as tumor size, grade, and lymph node status. However, multigene expression assays like MammaPrint offer additional prognostic information that may alter treatment recommendations. This study aimed to assess the level of agreement between MammaPrint-based genomic risk classification and chemotherapy recommendations derived from National Comprehensive Cancer Network (NCCN)-based clinical criteria in a cohort of Bosnia and Herzegovina breast cancer patients.

Methods

We retrospectively analyzed 66 patients with HR+/HER2-, node-negative early breast cancer treated between 2023 and 2024. All patients underwent MammaPrint testing, which classified tumors as either low risk or high risk for distant recurrence. Clinical risk was assessed using a simplified NCCN-guided algorithm, in which chemotherapy was recommended for tumors >2 cm and/or grade three histology. The primary outcome was the rate of concordance between genomic and clinical risk classifications. Statistical analysis included descriptive summaries, cross-tabulation, and Cohen’s kappa to evaluate agreement.

Results

Of the 66 patients analyzed, MammaPrint classified 27 (40.9%) as high risk and 39 (59.1%) as low risk. Based on NCCN criteria, 36 patients (54.5%) were considered clinically high-risk and recommended for chemotherapy, while 30 (45.5%) were not. Concordance between genomic and clinical classifications was observed in 37 patients (56.1%), while 29 patients (43.9%) showed discordant results. The most common discordance pattern was a clinically high-risk but genomically low-risk classification, observed in 19 cases (65.5% of discordant pairs). Cohen’s kappa for agreement between methods was 0.136, indicating slight agreement beyond chance. McNemar’s test yielded a χ² value of 10.0 (p = 0.036), suggesting significant asymmetry in discordance patterns.

Conclusion

This study highlights a substantial rate of discordance between MammaPrint genomic risk and NCCN-based clinical risk assessment. In our cohort, reliance on clinicopathological features alone would have led to different chemotherapy recommendations in over half of the cases. These findings support the clinical utility of multigene assays in refining adjuvant treatment decisions and reducing potential overtreatment in early breast cancer.

## Introduction

Early-stage breast cancer treatment decisions, particularly for hormone receptor-positive, HER2-negative tumors, rely on accurate risk stratification to balance the benefits of adjuvant chemotherapy against potential overtreatment. Traditional prognostic factors, patient age, tumor size, histologic grade, and lymph node status, form the backbone of clinical risk assessment​ [1]. However, many patients classified as high risk by these clinicopathological criteria may have excellent outcomes with endocrine therapy alone, meaning that a substantial subset could be overtreated with chemotherapy [2]. This recognition has driven the development of multigene expression assays that more precisely predict recurrence risk and chemotherapy benefit in early breast cancer​ [3]. Genomic assays such as the 21-gene Recurrence Score (Oncotype DX) and the 70-gene signature (MammaPrint) have become established tools for guiding adjuvant therapy in estrogen receptor-positive (ER+), HER2-negative disease [4]. Oncotype DX quantifies recurrence risk on a continuous scale and was validated in the Trial Assigning Individualized LOptions for Treatment (TAILORx) trial. TAILORx demonstrated that most women with intermediate oncotype scores (Recurrence Score 11-25) derive no significant benefit from chemotherapy; nine-year invasive disease-free survival was approximately 83-84% with endocrine therapy alone, virtually identical to chemoendocrine therapy outcomes​ [5]. Distant recurrence rates and overall survival were likewise equivalent, confirming that about 70% of ER+/HER2-, node-negative patients can be treated safely with endocrine therapy and avoid the toxicity of chemotherapy​ [5]. These findings reinforced that traditional factors alone often overestimate risk, and that genomic profiling can identify patients who can safely forgo chemotherapy. MammaPrint is a microarray-based 70-gene signature that stratifies early breast cancers into two categories: low risk or high risk of distant recurrence at 10 years. Early studies established the prognostic power of this assay; for example, a landmark analysis showed that the 70-gene “good prognosis” signature was a stronger predictor of 10-year metastasis-free survival in young breast cancer patients than standard histopathological criteria [6]. The clinical utility of MammaPrint was confirmed in the prospective Microarray In Node-negative and 1 to 3 positive lymph node Disease may Avoid ChemoTherapy (MINDACT) trial. In MINDACT, 6,693 patients had both clinical risk (based on Adjuvant! Online algorithms) and genomic risk assessed; 23% of patients had discordant results in terms of high clinical risk but low genomic risk. Among these clinically high-risk but genomically low-risk patients who were spared chemotherapy, the five-year distant metastasis-free survival was 94.7% (95% CI 92.5-96.2). This outcome was non-inferior to the chemotherapy arm ​indicating that omission of chemotherapy did not compromise short-term outcomes. Approximately 46% of women who would traditionally be recommended adjuvant chemotherapy did not actually require it when guided by a low-risk 70-gene signature​ [7].

As a result of these advances, international guidelines have incorporated multigene assays into risk stratification for early breast cancer. The American Society of Clinical Oncology (ASCO) and National Comprehensive Cancer Network (NCCN) guidelines endorse the use of assays like Oncotype DX and MammaPrint to inform adjuvant therapy decisions in ER+/HER2-, node-negative, and selected node-positive patients​ [8]. MammaPrint now carries level one evidence and consensus support for use in node-negative disease, reflecting its proven ability to impact clinical decision-making [9]. However, despite extensive global research, data from Bosnia and Herzegovina and the broader Southeast Europe region remain scarce, highlighting the novelty and importance of evaluating the utility of genomic risk assessment in this specific population. It is clinically relevant to determine how well genomic risk classifications align with traditional guideline-based recommendations in these populations, as differences could influence adoption of testing and optimize therapy decisions.

The present study addresses this gap by comparing MammaPrint genomic risk with NCCN guideline-based clinical risk assessment in a cohort of early breast cancer patients. Specifically, our aim is to evaluate whether genomic risk classification using MammaPrint can refine chemotherapy decisions beyond traditional NCCN criteria, thereby potentially reducing overtreatment and optimizing therapeutic choices. We retrospectively analyzed 66 women with ER-positive, HER2-negative, lymph node-negative breast cancer to examine how often the 70-gene signature would recommend against chemotherapy in patients who otherwise meet conventional criteria for chemotherapy, and vice versa. We hypothesize that significant discordance between these assessments will highlight the potential clinical utility of genomic profiling in more accurately guiding treatment decisions in our regional patient population.

## Materials and methods

Study design and patients

This study was a retrospective cohort analysis of female patients with early-stage breast cancer treated in Bosnia and Herzegovina. Eligible cases were identified from institutional databases between January 2023 and December 2024. Inclusion criteria were invasive carcinoma of the breast confirmed on histopathology; ER-positive status (with or without progesterone receptor expression); HER2-negative status; node-negative disease; and availability of a MammaPrint 70-gene signature result. Exclusion criteria included metastatic breast cancer at diagnosis, prior history of malignancy, previous chemotherapy or radiation therapy, and incomplete clinical or genomic risk data. All patients had operable tumors (pathological T1-T2, tumor size ≤5 cm confirmed by histopathological examination) and underwent definitive surgery (either breast-conserving surgery or mastectomy with sentinel lymph node biopsy). Standard clinicopathological data were collected retrospectively from electronic medical records and pathology reports, including patient age at diagnosis, tumor size, histological type, Nottingham histologic grade, presence of lymphovascular invasion, Ki-67 proliferation index, nodal status, ER and PR expression, and HER2 status. Histological grading was performed by experienced pathologists at our institution according to standardized Nottingham criteria, minimizing interobserver variability.

Risk assessment methods 

For each patient, two risk stratification approaches for adjuvant chemotherapy decision-making were compared: (i) genomic risk based on the MammaPrint 70-gene assay and (ii) clinical risk based on simplified NCCN guideline criteria. MammaPrint assays were conducted in a certified central reference laboratory to ensure standardized quality control and reproducibility. The genomic assay categorized tumors as low risk or high risk for distant recurrence. For the clinical risk assessment, we applied NCCN guideline-based criteria focusing on the tumor size and grade, given that all tumors were ER-positive and HER2-negative by design. Patients were categorized as high clinical risk (indicating a recommendation for adjuvant chemotherapy) if they had unfavorable pathological features traditionally associated with higher recurrence risk: specifically, tumor size >2 cm or histologic grade three. Patients with tumors ≤2 cm and grades one or two were considered low clinical risk, for whom adjuvant chemotherapy would not be routinely recommended in accordance with simplified NCCN recommendations. All patients included in this study were lymph node-negative. The Ki-67 proliferation index threshold of 20% was selected based on established international recommendations, including the St. Gallen consensus guidelines.

Statistical analysis 

Descriptive statistics (median with range and standard deviation, percentages, and 95% confidence intervals as appropriate) were used to summarize patient characteristics and risk categorization results. Agreement between genomic and clinical risk classification was quantified using Cohen’s kappa coefficient. McNemar’s test was employed to assess whether the proportion of discordant classifications significantly differed in one direction compared to the other. Logistic regression was performed to identify which clinical features were most strongly associated with discordant results between genomic and clinical risk assessments. Statistical significance was defined as a two-sided p-value <0.05. All statistical analyses were conducted using IBM SPSS Statistics for Windows, Version 25 (Released 2017; IBM Corp., Armonk, New York, United States).

## Results

**Figure 1:**
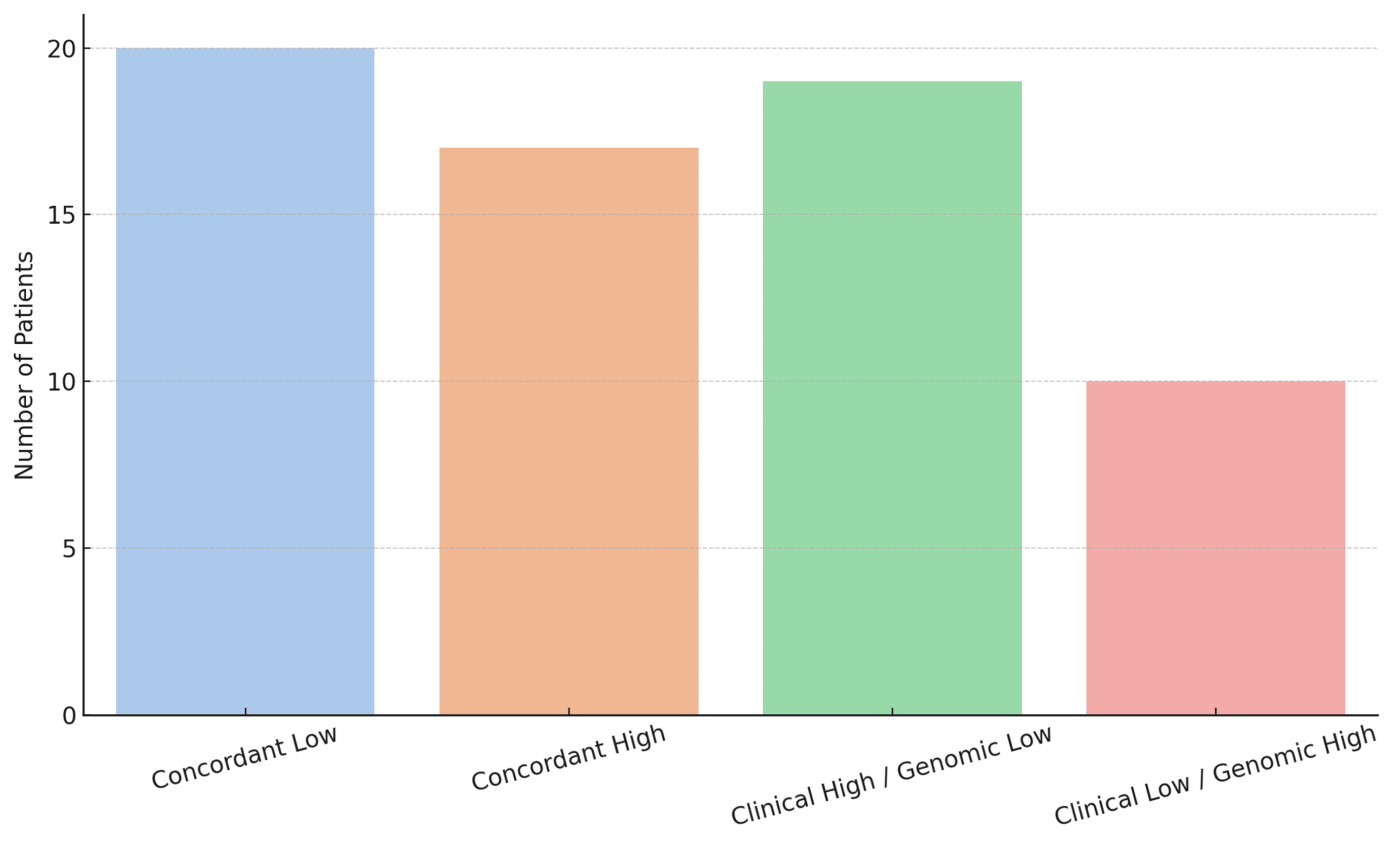
Bar chart distribution of concordant and discordant risk classifications between NCCN and MammaPrint NCCN: National Comprehensive Cancer Network

A total of 66 female patients diagnosed with HR+, HER2-negative, node-negative early breast cancer were included in the analysis. The mean age at diagnosis in the MammaPrint low-risk group was 53.8 ± 9.14 years (95% CI 50.8 - 56.9) and that in the MammaPrint high-risk group was 49.7 ± 8.42 years (95% CI 46.3-53). Tumors had a mean size of 1.81 cm ± 0.57 (95%CI 1.62-2.00) in the low-risk group and 1.97 cm ± 0.57 (95%CI 1.75-2.2) in the high-risk group. Histological grading revealed grade one in 10% of tumors, grade two in 53%, and grade three in 37%. Regarding the Ki-67 proliferation index, 66% of patients exhibited low proliferation (<20%), while 34% exhibited high proliferation (≥20%). Among patients with high-grade (grade 3) tumors, 33% were classified as low risk by MammaPrint. According to MammaPrint genomic testing, 27 patients (40.9%) were classified as high risk and 39 patients (59.1%) as low risk. By applying NCCN-based clinical criteria, chemotherapy was recommended for 36 patients (54.5%), whereas it was not recommended for 30 patients (45.5%) (Table 1). Agreement between the two risk classification methods occurred in 37 of 66 cases (56.1%). The discordance rate was 43.9%, with the predominant discordance pattern involving clinically high-risk but genomically low-risk classifications, observed in 19 (65.5%) of the discordant cases (Figure 1).

**Table 1:** Patient characteristics and MammaPrint risk classification ^a^Mann-Whitney U test; ^b^Chi-square test NCCN: National Comprehensive Cancer Network

Patient characteristics	MammaPrint results N = 66		
Age	Low risk (n=39)	High risk (n=27)	P-value 0.09^a^
Mean	53.8 years (±9.14 SD)	49.7 years (± 8.42 SD)	
Tumor size			0.33^a^
Mean	1.81 cm ± 0.57	1.97 cm ± 0.57	
Clinical risk (NCCN)			0.02^b^
Low risk	20	10	
High risk	19	17	
Tumor stage			0.495^b^
T1a	1	0	
T1b	5	1	
T1c	14	11	
T2	19	15	
Grade			0.04^b^
G1	5	2	
G2	21	14	
G3	13	11	
Tumor type			0.635^b^
Ductal	36	24	
Lobular	8	8	
Ki 67			0.111^b^
Low (<20%)	29	15	
High (≥20%)	10	12	

Cohen’s Kappa statistic was calculated to assess the agreement beyond chance between MammaPrint and NCCN criteria. The Kappa coefficient obtained was 0.136 (95% CI 0.064-0.365), indicating slight agreement beyond what could be expected by chance.

**Figure 2:**
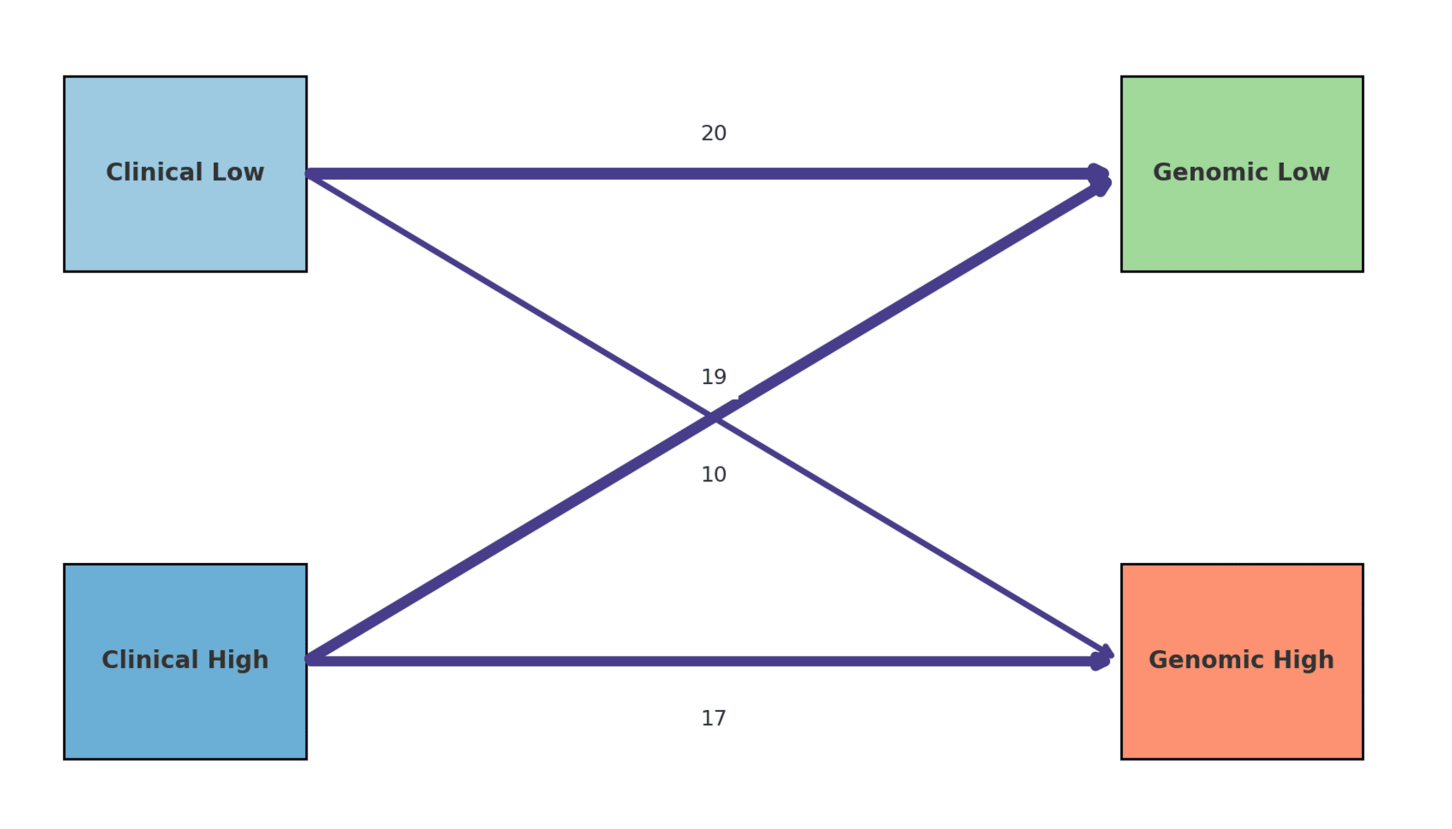
Sankey-style diagram of reclassification of patients between clinical and genomic risk categories

McNemar’s test was performed to evaluate symmetry in discordant results between MammaPrint and NCCN guidelines. The test yielded a χ² value of 10.0 (p = 0.036), suggesting significant asymmetry in discordance patterns, confirming that MammaPrint more frequently classified patients into a lower-risk category compared to the clinical assessment (Figure 2).

A univariate logistic regression model was built to identify clinical predictors associated with a discordance between MammaPrint and clinical assessment. Variables included in the regression model were age, tumor size, histological grade, and Ki-67 proliferation index.

In the logistic regression analysis, only histological grade emerged as a significant predictor of discordance (OR = 4.5; 95% CI: 2.77-4.86; p = 0.05). The tumor size also showed a trend toward significance (OR = 1.8; 95% CI: 1.2-2.3; p = 0.071), while the patient age and Ki-67 proliferation index did not reach statistical significance (p = 0.59 and p = 0.41, respectively) (Table 2).

**Table 2:** Logistic regression for the discordance in risk classification

Patient characteristics	OR	95% CI	p-value
Tumor size	1.8	1.2-2.3	0.07
Histological grade	4.5	2.77-4.86	0.05
Ki-67	1.2	0.86 - 2.2	0.41
Age	1.01	0.039 - 1.79	0.59

## Discussion

Nearly half of the patients were classified into different risk categories by genomic profiling versus traditional clinical-pathological criteria. This high discordance is consistent with prior studies. For example, an analysis with 10-year outcomes in patients with early breast cancer treated according to the heritability (PATH) dataset reported genomic vs. clinical risk discordance in ~42-43% of tumors​ [10]. Similarly, the landmark MINDACT trial noted that 46% of women who were clinically high-risk by Adjuvant! Online criteria were reclassified as low risk by MammaPrint​ [2]. Our data also mirror the predominant pattern of discordance observed in these larger studies: most discordant cases in our series were patients deemed high-risk by clinical factors but low-risk by the 70-gene signature. MINDACT highlighted this same imbalance, with a large subset of clinically high-risk patients showing indolent genomic profiles​. In contrast, the opposite pattern, clinical low risk but genomic high-risk, accounted for far less of discordant cases in our study, though these cases remain clinically important as discussed below.

The agreement between the two risk stratification methods was only slight. This aligns with previous reports demonstrating weak agreement between genomic assays and traditional risk models. One study found, in a cohort of clinically high-risk breast cancers, that 36% of tumors had discordant risk predictions between MammaPrint and another genomic test (EndoPredict), with a low inter-test correlation (κ = 0.27)​ [11].

Although the study compared two different gene signatures, it underscores the broader point that different modalities of risk assessment often classify patients differently. In our case, the discordance between NCCN clinical criteria and the 70-gene signature underscores that these tools are capturing distinct facets of tumor biology.

Notably, our finding that most discordant cases were clinical high/genomic low has critical clinical implications. Overtreatment is a major concern in such scenarios. By clinical criteria alone, these patients would traditionally be recommended adjuvant chemotherapy, exposing them to toxicity and psychosocial burden, despite having tumors with favorable biology that are unlikely to recur. The MINDACT trial provides reassuring prospective evidence for foregoing chemotherapy in this group: at five-year follow-up, patients who were clinically high-risk but genomic low-risk and who did not receive chemotherapy had a distant metastasis-free survival of 94-95%​. In the updated eight-year analysis of MINDACT, the distant metastasis-free survival for this subgroup remained ~95% at five years (95.1%, 95% CI 93.1-96.6%), ​confirming the excellent outcomes with endocrine therapy alone [12]. 

Another study observed in TAILORx showed that there were no distant recurrences at nine years among women who had high clinical risk features but a low Oncotype recurrence score (0-10) and were treated with endocrine therapy alone​. On the other hand, patients with clinically low risk but genomically high-risk represent cases of potential undertreatment if genomics are ignored. MINDACT sheds light on this scenario as well: although it was not the primary focus, MINDACT reported that clinical low-risk/genomic high-risk patients had a ~5% risk of distant metastasis at five years despite all standard treatments, a risk level that was considered borderline and not low enough to confidently omit chemotherapy.​ Our data emphasize that purely clinicopathologic assessment can miss a subset of biologically high-risk cancers which is precisely the rationale for incorporating genomic assays into practice. It is also worth noting that in TAILORx, younger women under 50 with intermediate Oncotype scores benefited from chemotherapy particularly if certain clinical features were adverse​ [13,14].

A high histologic grade is well known to correlate with more aggressive tumor biology and indeed with higher genomic risk scores in assays like Oncotype DX and MammaPrint​ [15]. We found that grade three tumors were significantly associated with discordant classifications, a finding consistent with prior studies which reported approximately one-quarter of clinically high-risk, grade three tumors as MammaPrint low-risk [16]. Recent studies using different genomic assays and prognostic models, including a Chinese cohort and an EndoPredict-based assessment, also consistently reported substantial clinical-genomic discordance, particularly for high-grade tumors [17,18]. Clinicians should thus be aware that high-grade tumors may not always correlate with genomic high-risk profiles.

Our findings are particularly relevant for resource-limited settings, where routine genomic testing may not yet be widely available. In such settings, recognizing clinical features frequently associated with genomic discordance, such as high tumor grade, can help guide more nuanced decision-making until broader genomic testing becomes feasible.

Taken together, the literature to date, including our current study, consistently shows that relying on clinicopathologic features alone can lead to misclassification in a substantial minority of cases. By incorporating genomic assays, we obtain a more nuanced risk assessment that often reclassifies patients into a different risk category, with direct implications for therapy.

Limitations

This study has several limitations that should temper the interpretation of our results. First, the sample size is relatively small. While our findings are descriptive and in line with larger studies, the statistical power is limited, especially for subgroup analyses. Second, our analysis is retrospective and subject to inherent biases. Third, the clinical risk classification was based on NCCN guideline criteria, which, while standardized, is not a single numeric tool but rather a composite of recommendations. Finally, the lack of long-term clinical outcome data precludes assessment of actual recurrence risk and survival implications. Prospective, multicenter studies with larger cohorts and long-term follow-up are required to validate our findings.

## Conclusions

This study demonstrates that a substantial proportion of early-stage, hormone receptor-positive, HER2-negative, node-negative breast cancer patients exhibit discordance between clinical and genomic risk classifications, particularly with a tendency for MammaPrint to downgrade risk in clinically high-risk cases.

These findings support the utility of genomic profiling in refining adjuvant chemotherapy decisions, helping to avoid both overtreatment and undertreatment. Incorporating MammaPrint into multidisciplinary decision-making may enhance personalized treatment strategies and improve the quality of care. Further studies with larger cohorts and long-term follow-up are warranted to validate these observations and assess their impact on patient outcomes.
